# The intracellular cation channel TMEM176B as a dual immunoregulator

**DOI:** 10.3389/fcell.2022.1038429

**Published:** 2022-10-20

**Authors:** Marcelo Hill, Sofía Russo, Daniela Olivera, Mateo Malcuori, Germán Galliussi, Mercedes Segovia

**Affiliations:** ^1^ Laboratory of Immunoregulation and Inflammation, Institut Pasteur de Montevideo, Montevideo, Uruguay; ^2^ Immunobiology Department, Faculty of Medicine, University of the Republic, Montevideo, Uruguay; ^3^ Laboratory of Vascular Biology and Drug Development, Institut Pasteur de Montevideo, Montevideo, Uruguay

**Keywords:** immunoregulation, antigen presentation, inflammasomes, Tmem176b, ion channels

## Abstract

Characterizing immune regulatory pathways is critical to understand physiological and pathophysiological processes as well as to identify novel immunotherapeutic targets. The cation channel TMEM176B has emerged in the last years as a potential new immunoregulatory player and pharmacological target. Here, we review how expression data, clinical associations of genetic variants and functional studies support a dual role for TMEM176B in regulating immune responses. Thus, TMEM176B can inhibit effector immune responses in some settings whereas it may also promote immunity by supporting antigen presentation in others. We also discuss a potential role for TMEM176B in regulating type 2 and 3 immunity and comment recent data on modulation of DC biology and inflammasome activation as well as CD8^+^ T cell responses. Understanding the role of TMEM176B in immunity is critical to propose rational pharmacological approaches targeting this channel.

## Introduction

The immune system plays a key role in maintaining systemic homeostasis. To do so, immune responses aim at eliminating internal and external threats without damaging healthy tissues and organs. Such a challenging task needs to be controlled by cellular and molecular mechanisms that trigger the right response at the right place and for the right time. Antigens can trigger either effector players aiming at eliminating them or regulatory strategies that rather protect the triggers (Mellor and Munn, 2008). Protective responses result from tight regulation of effector and regulatory arms. In scenarios such as infections and cancer, effector responses are needed whereas regulatory players may be harmful. On the other hand, in pregnancy, transplantation and autoimmunity, regulatory responses will pave the way for homeostasis whereas effector pathways will lead to pathology. Moreover, immunotherapeutic interventions can target immune players to restore the stationary state. Thus, a deep characterization of immune-regulatory strategies is of paramount importance to understand physiology and pathology as well as to rationally develop immunotherapeutic approaches.

The characterization of immune checkpoints such as CTLA-4 and PD-1 has clearly shown that understanding immune regulation at the physiological level can impact on therapy at the clinics. Immune checkpoints can be defined as powerful molecular players that inhibit effector immune responses to prevent damage to healthy tissues. Accordingly, mice deficient in *Pdcd1* (Nishimura et al., 1999, 2001; Okazaki et al., 2003) and *Ctla4* (Tivol et al., 1995; Waterhouse et al., 1995) suffer from devastating autoimmunity. In humans, blockade of CTLA-4 and PD-1 triggers strong anti-tumoral immune responses in some patients, also increasing the odds of suffering from immune-related adverse events (Ribas and Wolchok, 2018). In complement to immune checkpoints, other immune regulatory strategies, which are also potential therapeutic targets, are needed to achieve homeostasis. In contrast to immune checkpoints, other immune regulators can work as two-way switches and in some circumstances control effector mechanisms at different degrees or even promote them. This dual behavior has been reported for highly relevant immune players such as IL-2 (Pol et al., 2020) and type 1 and 2 interferons (Lee and Ashkar, 2018). In this context, it may be more difficult to anticipate a functional outcome when intervening the pathway. However, its rational modulation may lead to a more nuanced effect and thus, potentially uncoupling protective effector immune responses and immune-mediated damage. Nevertheless, a deep knowledge of the pathway is needed to develop rational immune interventions to avoid unexpected outcomes.

### TMEM176B as a new player in the landscape of immune regulation

TMEM176B is an intracellular, acid-sensitive, non-specific cation channel (Segovia et al., 2014) which has been linked to the membrane-spanning 4A (MS4A) family (Louvet et al., 2005; Segovia et al., 2014; Eon Kuek et al., 2016; Mattiola et al., 2021). TMEM176B shares 30% identity with TMEM176A, another member of the MS4A family. These are ubiquitous proteins highly expressed in primary and secondary lymphoid organs, colon, lung and liver (Louvet et al., 2005; Condamine et al., 2010). Within leukocytes, it is highly expressed by the myeloid compartment, although expression by RORγT^+^ cells such as ILC3, Th17 and γδ T cells has been described (Drujont et al., 2016). Early studies by Cuturi and others have shown that in rat allotransplantation models *Tmem176b* expression was associated with immunological tolerance, suggesting a potential immunoregulatory role for this protein (Louvet et al., 2005). Accordingly, high TMEM176B expression in the tumor infiltrate in human colon cancer correlates with poor survival (Segovia et al., 2019). *TMEM176B* expression in tumor biopsies has also been associated with resistance to immune checkpoint blockers in melanoma patients (Segovia et al., 2019). On the other hand, *TMEM176B* expression in peripheral blood has been associated with clinical responses to anti-TNF therapy in rheumatoid arthritis (Wysocki and Paradowska-Gorycka, 2022). In multiple sclerosis, TMEM176B has been reported as a member of the gene expression signature (Nickles et al., 2013). In particular, the A134T genetic variant of TMEM176B could be associated with multiple sclerosis although this has not been formally proven. (Nickles et al., 2013). Moreover, this genetic variant has been linked to beneficial prognosis in colorectal cancer (Cambui et al., 2022). In the last years we started to get some insight on the potential role of this molecule in immunoregulatory mechanisms. Here we review the literature supporting a potential dual role for TMEM176B in regulating immunity.

### TMEM176B expression in DCs and RORγT^+^ cells

To understand the functional role played by TMEM176B in the immune system, characterizing its cellular expression and subcellular localization is critical. Within rat leukocytes*, Tmem176b* was shown to be highly expressed by the myeloid cell compartment (Louvet et al., 2005). Specifically, high *Tmem176b* expression at the mRNA level was detected in rat peritoneal macrophages and in splenic CD103^+^ (OX62) dendritic cells. Within those cells, *Tmem176b* was expressed at higher levels in the CD4^+^ subset (rat DCs thought to resemble type 2 conventional DCs) *versus* CD4^−^ cells. Accordingly, immunofluorescence studies in the spleen showed that TMEM176B staining co-localized with MHC class II and CD172 as well as with CD68 (Louvet et al., 2005). In mice, *Tmem176b* was expressed by both type 1 and type 2 cDCs, although expression levels were higher in the latter (Condamine et al., 2010; Crozat et al., 2011; Segovia et al., 2014). In tumor-draining lymph nodes, *Tmem176b* expression in cDC2 was shown to be supported by regulatory T (Treg) CD4^+^ cells (Binnewies et al., 2019), suggesting an immunoregulatory role for this cation channels in cDC2. Moreover, *Tmem176b* expression was associated with regulation of immune responses by migratory DCs (Anandasabapathy et al., 2014).

Recently, transcriptomic studies at the single-cell level have revealed an increasing complexity of leukocyte subsets. Particularly, although cDC1 (XCR1^+^) are thought to represent a homogeneous population, cDC2 (CD1C^+^) have been shown to gather different subsets in mice and humans. In human healthy blood, Villani et al. have identified two subpopulations within CD14^−^ CD1C^+^ DCs, DC2 and DC3 (Villani et al., 2017). DC3 are CD11C^lo^, CD1C^lo^, CD163^+^, CD36^+^ whereas DC2 are CD11C^+^, CD1C^+^, CD32B^+^. DC3 differentially expressed inflammatory genes such as inflammasome-related genes as well as *TMEM176B*. Functionally, both subsets stimulated the proliferation of allogeneic CD4^+^ and CD8^+^ T cell proliferation *in vitro* (Villani et al., 2017). Dutertre et al. then added more phenotypic, functional and molecular heterogeneity to cDC2s (Dutertre et al., 2019). They showed that human cDC2 comprise CD5^+^ DC2 and CD5^−^ DC3. Within the DC3 compartment, they described three clusters: CD163^-^, CD163^-^ CD14^+^ and CD163^+^ CD14^+^. CD163^+^ CD14^+^ DC3 expressed high levels of inflammasome-related genes (Dutertre et al., 2019). CD1C^+^ CD163^+^ DC3s develop in a GM-CSF-dependent pathway and have been identified as immediate precursors of CD14^+^ CD1C^+^ CD163^+^ FcεRI^+^ inflammatory DCs (Bourdely et al., 2020). In cells stimulated with Toll-like receptors agonists (TLRs), *TMEM176B* was differentially expressed in DC3 *versus* cDC2 (Bourdely et al., 2020). Functionally, stimulated DC3 secreted higher levels of TNF and IL-1β as compared to cDC2. Furthermore, DC3 were able to prime naïve CD4^+^ and CD8^+^ T cells *in vitro*. Moreover, DC3 efficiently trigger CD103 expression in CD8^+^ T cells, which is a hallmark of tissue resident memory (TRM) cells. Inflammatory DC3 were expanded in the blood of systemic lupus erythematosus patients and correlated with disease activity (Dutertre et al., 2019). Accordingly, TMEM176B emerges as a potential target to control the inflammatory activity of DC3 cells in autoimmunity settings. Thus, unraveling the role of TMEM176B in DC3 is critical to understand its physiological and pathological impact.

It has been classically accepted that subsets of cDC specialize in the activation of particular T cell populations. Thus, cDC1 initiate CD8^+^ T cell responses whereas cDC2 prime CD4^+^ T cells (Dudziak et al., 2007). Nevertheless, the DC3 subset of cDC2 has been shown to activate CD4^+^ and CD8^+^ T cells (Villani et al., 2017; Bourdely et al., 2020). Human CD14^+^ CD163^+^ DC3 have been shown to specifically trigger Th2 and Th17 differentiation in comparison to CD5^+^ DC2, CD14^−^ CD163^-^ and CD14^+^ CD163^-^ DC3. Moreover, lung cDC2 have been shown to promote differentiation of Th2 or Th17 cells depending on their maturation stage (Izumi et al., 2021). Thus, TMEM176B which is associated with the immature stage of DCs may regulate Th2 and/or Th17 differentiation in these subsets. In fact, *Tmem176b* was also found to be expressed in a Th2-driving CD11c^+^ PD-L2^+^ IRF-4^+^ CD301b^+^ DC subset in murine skin-draining lymph nodes (Gao et al., 2013). However, the functional role of Tmem176b in that subset and in type 2 immune responses remains to be elucidated.

In the tumor-draining lymph node of mice, *Tmem176b* deletion was associated with increased Caspase-1 activation in CD11b^+^ cDCs (cDC2) as well as with increased CD4^+^ RORγT^+^ cells *versus* WT animals (Segovia et al., 2019). *In vitro* restimulation of lymph node cells with tumoral antigens triggered IL-17 production in cells from *Tmem176b* ^*−/−*^ but not WT mice (Segovia et al., 2019). Thus, Tmem176b expression in cDC2/DC3 may control differentiation of Th17 cells.

TMEM176B was also shown to be expressed in RORγT^+^ cells such as human and mouse ILC3, Th17 and γδ T cells (Drujont et al., 2016). In human and mouse cDC2, TMEM176B expression has been associated with a subset of proinflammatory RORγT^+^ cells (Brown et al., 2019). Strikingly, *Tmem176b* was identified as one of a few genes that were dependent on RORγT in Th17 cells (Ciofani et al., 2012). Thus, *Tmem176b* may help to determine the differentiation program in Th17 cells. However, the biological role of intrinsic TMEM176B in RORγT^+^ cells remain elusive. In certain circumstances, Th17 cells can ensemble inflammasomes leading to Caspase-8 activation (Martin et al., 2016; Zhang et al., 2022). Further work is needed to determine whether Tmem176b may control inflammasome activation and/or other relevant processes in Th17 cells.

### Control of DC biology by TMEM176B

TMEM176B is highly expressed by mouse bone marrow-derived DCs (BMDCs) and by human monocyte-derived DCs (MoDCs) (Louvet et al., 2005). Treatment with CD40L and TNF plus poly I: C down-regulated *TMEM176B* expression in rat splenic DCs and human Mo-DCs respectively (Louvet et al., 2005). Functionally, *Tmem176b* over-expression in BMDCs inhibited basal expression of MHC class II and CD86 and after LPS treatment (Louvet et al., 2005). In mouse BMDCs, *Tmem176a* and *Tmem176b* expression was down-regulated by LPS or poly I:C treatment (Condamine et al., 2010). Moreover, knock-down of TMEM176B or TMEM176A was associated with increased CD40, CD80, and CD86 expression as well as with increased allostimulatory capacity on lymph node cells (Condamine et al., 2010). In contrast, phenotypic analysis of *Tmem176b* ^*−/−*^ BMDCs showed no differences with WT cells (Segovia et al., 2014).

**FIGURE 1:**
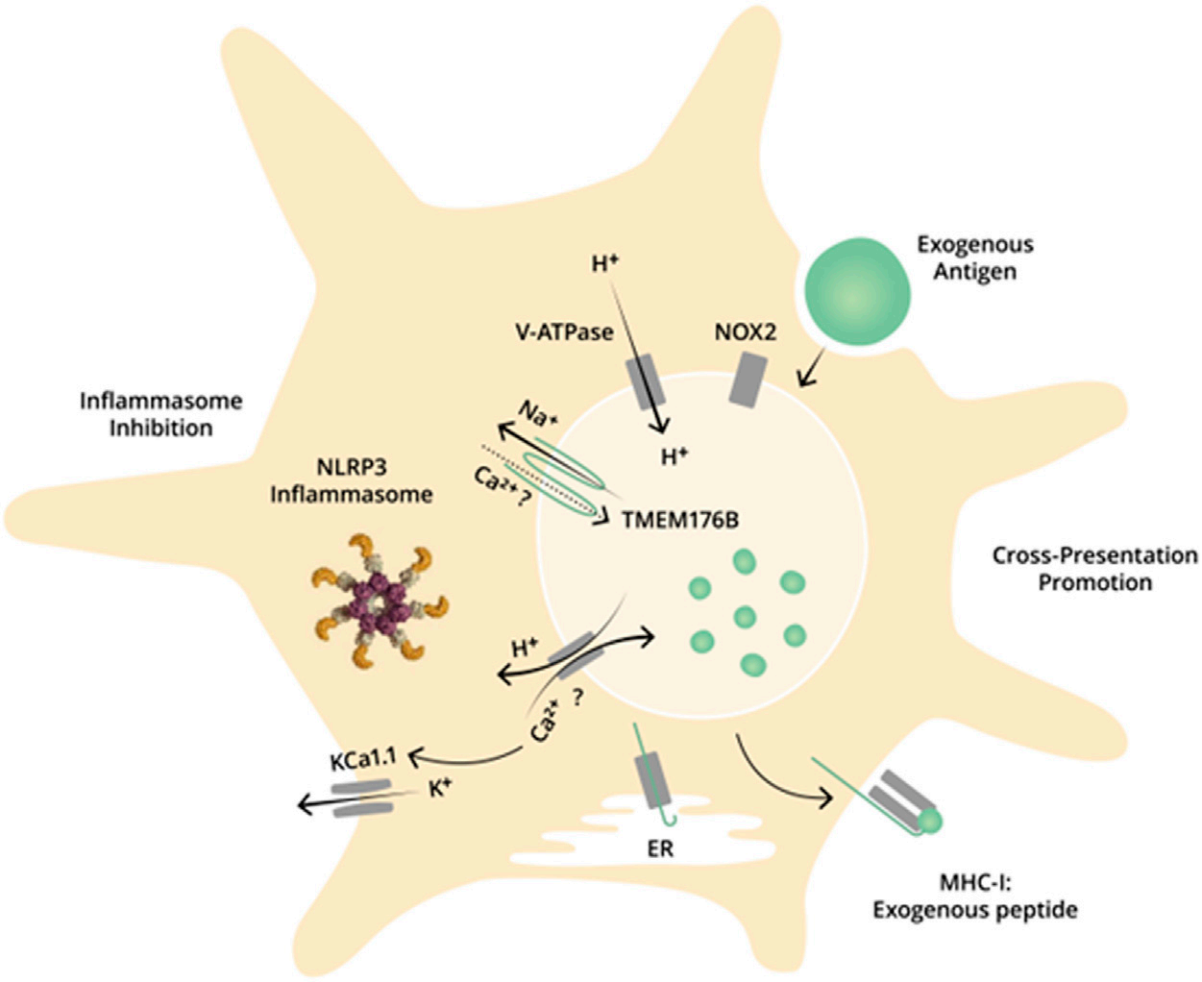
Dual role of TMEM176B in DCs: promotion of antigen cross-presentation and inhibition of inflammasome activation. TMEM176B at the endophagosmal membrane in DCs. TMEM176B exports Na^+^ from the endophagosomal lumen to the cytosol. This conductance alleviates the electrochemical gradient to V-ATPase, promoting the pumping of H^+^ into the lumen. A neutral pH in the endophagosomal lumen in DCs is critical to process antigens through the cross-presentation pathway. Accordingly, deletion of *Tmem176b* is associated with diminished V-ATPase activity, alkalinized phagosomal pH and impaired antigen cross-presentation. Moreover, TMEM176B inhibits NLRP3 inflammasome activation through ionic mechanisms. *Tmem176b* deletion is associated with KCa1.1-dependent inflammasome activation. TMEM176B controls cytosolic Ca^2+^ levels although the mechanisms of this effect remain unknown. Ca^2+^ might be internalized *via* TMEM176B into the phagosomal lumen and/or TMEM176B activity may promote H^+^/Ca^2+^ interchange, leading to decreased cytosolic Ca^2+^.

Determining the subcellular localization of TMEM176B has been critical to add functional data on the role of this protein in DCs. TMEM176B has been shown to be localized at the Golgi apparatus (Drujont et al., 2016; Lancien et al., 2021) and at endosomes and phagosomes in human and mouse DCs (Segovia et al., 2014). Given that TMEM176B belongs to a family (MS4A) that includes ion channels, we speculated that this protein may regulate endophagosomal pH. Indeed, we have shown that TMEM176B can transport cation channels across biological membranes. Moreover, endophagosomal pH is tightly regulated in DCs through the activity of vacuolar ATPase (V-ATPase) and NADPH oxidase 2 (NOX2) (Savina et al., 2006). We showed that *Tmem176b* ^*−/−*^ BMDCs display a transitory alkalinized phagosomal pH in comparison to WT cells (Segovia et al., 2014). Moreover, Na^+^ substitution with the membrane-impermeable molecule NMDG in the culture media, alkalinized phagosomes in WT but not in *Tmem176b* ^*−/−*^ BMDCs. These results suggested that TMEM176B might be responsible for a counterion conductance that may support V-ATPase activity. Accordingly, V-ATPase inhibition with Bafilomycin-1 alkalinized phagosomes in WT but not in *Tmem176b* ^*−/−*^ BMDCs (Segovia et al., 2014). This result, together with the alkalinized phagosomal pH, suggest that V-ATPase is inhibited in *Tmem176b* ^*−/−*^ BMDCs. Thus, we proposed that a *Tmem176b*-dependent counterion conductance supports V-ATPase activity leading to phagosomal pH regulation (Figure 1).

Antigen presentation to CD8^+^ T cells through the cross-presentation pathway strongly depends on neutral phagosomal pH within DCs. In fact, processing of different antigens was altered in *Tmem176b* ^*−/−*^ DCs, leading to deficient cross-presentation ability of those cells in comparison to WT ones. *In vivo*, the diminished antigen processing capacity of *Tmem176b* ^*−/−*^ tolerogenic BMDCs was associated with reduced numbers of CD8^+^ Treg cells and shorter allograft survival in comparison to animals injected with WT tolerogenic BMDCs. It has also been suggested that genetic deletion of both *Tmem176b* and *Tmem176a* may affect antigen processing through the MHC class II pathway in cDC2 (Lancien et al., 2021). Thus, antagonism of both molecules may inhibit antigen presentation and effector immune responses in some circumstances although Lancien et al. did not succeed in finding significant differences in EAE severity when comparing WT and *Tmem176a* ^*−/−*^ *Tmem176b* ^*−/−*^ animals (Lancien et al., 2021).

### TMEM176B-dependent intracellular ion transport

Characterizing TMEM176B as an ion channel has been a milestone in the research targeting this molecule. Understanding its molecular function has been key to identify inhibitory and activating compounds (Segovia et al., 2019; Duhalde Vega et al., 2022) and has the potential to help understand the functional impact (gain or loss of function) of clinically relevant genetic variants. Different lines of evidence led us to propose the hypothesis that TMEM176B may be an ion channel. Its localization in endophagosomal membranes (where pH is tightly regulated) together with significant impacts on excitable tissues supported this possibility. Specifically, transgenic rats over-expressing TMEM176B showed a prominent vacuolar disease in skeletal muscle (Cuturi and others, unpublished). Moreover, a line of *Tmem176b* ^*−/−*^ mice was shown to suffer from cerebellar ataxia in two-thirds of animals (Maeda et al., 2006). This defect was associated with a severe loss of granule neurons in the cerebellum. Moreover, other members of the MS4A family such as CD20 and MS4A2 are bona fide ion channels (Mattiola et al., 2021). Thus, in collaboration with the electrophysiologist Pierre Charnet we showed that TMEM176B overexpression in *Xenopus* oocytes was associated with an acid-sensitive, slow inward conductance (Segovia et al., 2014). Substitution of extracellular Na^+^ with NMDG as well as a reversal potential near 0V helped us to determine that TMEM 176B was a non-selective cation channel (Segovia et al., 2014). TMEM176B physically interacts with TMEM176A (Condamine et al., 2010; Cuajungco et al., 2012) and over-expression of both proteins triggers a conductance with greater amplitude than the ones obtained with each molecule separately (Drujont et al., 2016). However, we lack information on the stoichiometry as well as potential functional differences of homo and hetero channels. TMEM176A may also interact with other channels such as TRPML2 (Cuajungco et al., 2014).

TMEM176B-dependent ion transport in phagosomes certainly impacts on cytosolic processes. Inflammasome activation critically depends on the cytosolic content of cations and anions (Gong et al., 2018). Inflammasomes are cytosolic multiprotein complexes that sense cellular stress and lead to Caspase-1-dependent activation of IL-1β and IL-18 (Rathinam and Fitzgerald, 2016). *Tmem176b* ^*−/−*^ DCs secrete larger quantities of IL-1β and IL-18 than WT cell when stimulated with NLRP3 triggers such as ATP, nigericin and alumina particles. Accordingly, TMEM176B over-expression significantly blocked IL-1βsecretion triggered by nigericin in human THP-1 macrophages. *In vivo*, i. p ATP injection triggered enhanced recruitment of peritoneal neutrophils in *Tmem176b* ^*−/−*^ *versus* WT animals. *Casp1* deletion in *Tmem176b* ^*−/−*^ mice completely blocked neutrophils recruitment, showing that the observed enhanced recruitment in *Tmem176b* ^*−/−*^ mice was inflammasome-dependent. In human primary monocytes, pharmacological activation of TMEM176B with the flavonoid isoquercetin inhibited IL-1β secretion and Caspase-1 activation triggered by SARS-CoV-2 (Duhalde Vega et al., 2022). Moreover, TMEM176B overexpression completely blocked IL-1β secretion and Caspase-1 activation triggered by the ion channel SARS-CoV-2 envelope (E) protein (Duhalde Vega et al., 2022). TMEM176B therefore inhibits inflammasome activation, probably through ionic mechanisms (Figure 1). In fact, *Tmem176b* ^*−/−*^ BMDCs showed enhanced cytosolic Ca^2+^ *versus* WT cells when stimulated with ATP. Accordingly, cytosolic Ca^++^ chelation with BAPTA-AM completely blocked IL-1β secretion in WT and *Tmem176b* ^*−/−*^ BMDCs (Segovia et al., 2019). Furthermore, blockade of K^+^ efflux also inhibited IL-1βsecretion in WT and *Tmem176b* ^*−/−*^ BMDCs. Accordingly, IL-1β secretion in *Tmem176b* ^*−/−*^ BMDCs was highly sensitive to blockade of the Ca^2+^-activated K^+^ channels KCa3.1 (Segovia et al., 2019), which are involved in inflammasome activation (Eugenia Schroeder et al., 2017). Thus, TMEM176B may inhibit inflammasome activation by controlling cytosolic Ca^++^. Nevertheless, the intimate mechanisms of this effect remain unknown.

### TMEM176B and CD8^+^ T cell responses: *Je t’aime, moi non plus*

Inflammasome activation can impact on adaptive immune responses and anti-tumoral immunity (Segovia et al., 2019; Hill et al., 2020; Segovia et al., 2020). In mouse experimental models, genetic deletion of *Tmem176b* was associated with controlled tumor progression in EG7 lymphoma, MC38 colon and LL2 lung cancer. Moreover, anti-tumoral CD8^+^ T cell responses were strongly reinforced in *Tmem176b* ^*−/−*^ *versus* WT animals. In fact, depletion of the CD8 compartment abolished the protective effect observed in *Tmem176b* ^*−/−*^ mice. Mechanistically, both tumor control and anti-tumoral CD8^+^ T cell responses depended on enhanced inflammasome activation in *Tmem176b* ^*−/−*^ mice since they were reversed by IL-1β blockade and *Casp1* deletion. Furthermore, the TMEM176B inhibitor BayK8644 controlled tumor growth in a *Tmem176b*, *Casp1/11* and CD8-dependent manner. BayK8644 also improved the anti-tumor efficacy of immune checkpoint blockers in EG7 lymphoma, 5,555 melanoma and MC38 colon cancer. Nevertheless, BayK8644 monotherapy failed to control the growth of established tumors. The dual role played by TMEM176B, promoting antigen cross-presentation while inhibiting inflammasome activation, may help to understand the limited therapeutic efficacy of TMEM176B blockers as well as controversial results recently reported on the role of TMEM176B in regulating anti-tumoral CD8^+^ T cells. In fact, CD8^+^ T cells recognize tumoral antigens in the context of MHC I molecules through the cross-presentation pathway (Alloatti et al., 2017) and, as commented above, we have shown that Tmem176b promotes antigen cross-presentation by controlling phagosomal acidification in DCs (Segovia et al., 2014). Louvet and others have failed to observe differences in tumor progression when comparing EG7 growth in WT and *Tmem176b* ^*−/−*^ mice (Lancien et al., 2021). However, since only 40% of the injected WT mice developed tumors, these results should be taken with caution. In contrast, Jiang et al. reported that in B16 melanoma, tumor growth was accelerated in *Tmem176b* ^*−/−*^ mice in association with diminished tumor infiltration by CD8^+^ T cells in comparison to WT animals (Jiang et al., 2022). Although further work is needed to understand this controversy, decreased antigen cross-presentation capacity in *Tmem176b* ^*−/−*^ mice may explain these observations. Thus, in different models, the impact of inflammasome activation on CD8^+^ T cell responses may be different (Segovia et al., 2019; Tengesdal et al., 2021). In tumors where CD8^+^ T cell responses are reinforced by enhanced inflammasome activation, *Tmem176b* deletion may generate anti-tumoral immune responses despite compromised cross-presentation. In contrast, in tumors where inflammasome activation does not play a relevant role in anti-tumoral immunity, deficient antigen cross-presentation in *Tmem176b* ^*−/−*^ animals may lead to poor tumor control. However, formulation of TMEM176B blockers may help to uncouple its dual roles in innate and adaptive anti-tumoral immunity. Moreover, the limited therapeutic efficacy of TMEM176B blockers may be linked to its potential capacity to inhibit antigen cross-presentation. We observed that BayK8644 does inhibit the processing of antigens through the cross-presentation pathway in mouse splenic DCs *in vitro* (Victoria et al., 2022). In cross-presentation, phagosomal antigen processing occurs through a fast kinetic. In fact, a time window of 25 min has been proposed within which antigens must be processed (Howland and Wittrup, 2008). Thus, exogenous antigens that were not processed before that time, would not be cross-presented (Howland and Wittrup, 2008). We therefore speculated that formulating BayK8644 through a strategy that allows a slow release kinetic in endosomes may prevent inhibition of cross-presentation by the compound while potentially maintaining its capacity to induce inflammasome activation. We recently showed that formulating BayK8644 in slow-releasing nanoparticles (NPs) triggered inflammasome activation while preventing the inhibition of antigen cross-presentation and led to improved anti-tumoral efficacy of the compound (Victoria et al., 2022). The therapeutic effect was associated with reinforced tumor infiltration by total and tumor-specific CD8^+^ T cells.

Furthermore, in coronavirus disease TMEM176B was reported to play a protective role by controlling inflammasome-dependent T cell dysfunction. In COVID-19 patients, TMEM176B expression in peripheral blood and bronchioalveolar lavages was associated with mild disease (Duhalde Vega et al., 2022; Pekayvaz et al., 2022). In mice infected with the β-coronavirus Murine Hepatitis Virus (MHV)-A59, *Tmem176b* ^*−/−*^ mice showed significantly higher viral loads and diminished survival *versus* WT animals in an inflammasome-dependent manner. Enhanced inflammasome activation in *Tmem176b* ^*−/−*^ mice also led to undermined anti-viral CD8^+^ T cell responses due to exhaustion. Exhausted T cells are a specific lineage of dysfunctional cells characterized by impaired effector mechanisms and transcriptional, epigenetic and metabolic programs as well as the expression of inhibitory receptors such as programmed cell death 1 (PD-1) (Blank et al., 2019; McLane et al., 2019). Therapeutically, T cell exhaustion can be modulated by blocking PD-1 and its cognate ligand PD-L1 (Barber et al., 2006). Accordingly, anti-PD-1 therapy significantly improved viral load and survival in *Tmem176b* ^*−/−*^ but not in WT animals. In critical COVID-19 patients, low monocytic expression of TMEM176B was associated with increased plasmatic active Caspase-1 which also correlated with CD8^+^ T cell exhaustion. Moreover, TMEM176B-dependent cross-presentation may also protect the host from critical coronavirus disease. Differential links between inflammasome activation and subsets of exhausted CD8^+^ T cells in cancer and coronavirus disease may also help to understand the complex relationship between TMEM176B and CD8^+^ T cell responses.

## Concluding remarks

TMEM176B arises as an emergent immunoregulatory player. It is possible that this ion channel inhibits immune effector cells in some circumstances whereas in other settings may promote them, particularly by enhancing antigen presentation to T lymphocytes. We therefore need to answer different questions to increase the odds of targeting this molecule in clinical settings. Particularly, characterization of regulatory partners controlling TMEM176B expression/activity as well as immune regulators controlled by TMEM176B may add critical information. Finally, the role played by TMEM176B in type 2 and type 3 immunity as well as in different inflammatory settings will certainly be unraveled in the next years.
